# Living with and caring for somatic symptom disorders: a qualitative interview-study with patients and GPs using a deductive-inductive content analytic approach

**DOI:** 10.1186/s12888-026-08750-2

**Published:** 2026-10-13

**Authors:** N. J. Pohontsch, M. Lehmann, T. Zimmermann, B. Löwe, M. Scherer

**Affiliations:** 1 Department of General Practice and Primary Care, University Medical Center Hamburg-Eppendorf, Martinistr. 52, 20246 Hamburg, Germany; 2 Department of Psychosomatic Medicine and Psychotherapy, University Medical Center Hamburg-Eppendorf, Martinistr. 52, 20246 Hamburg, Germany

**Keywords:** General practitioners, Qualitative research, Somatic symptom disorders, Primary health care, Psychosomatic medicine

## Abstract

**Background:**

Persistent physical symptoms are common in general practice and often occur as part of a somatic symptom disorder. Patients suffering from somatic symptom disorders and their general practitioners (GPs) often experience barriers to mutual trust and effective care. Frequently, studies using a one-sided perspective bear mutual accusation about shortcomings of the respective other side. This study examines corresponding perspectives of patients with somatic symptom disorders and their treating GPs to capture their views on individual patient histories and thereby identify barriers to and potentially helpful aspects of communication, detection, care for and live with somatic symptom disorders.

**Methods:**

Qualitative face-to-face interviews were conducted with dyads of patients with somatic symptom disorders and their treating GPs. The interviews focused on the patients’ medical history as well as barriers and facilitators of management and care in general practice. Patients and their respective GPs were interviewed separately using a semi-structured interview guide. Interviews were digitally recorded and transcribed verbatim. Transcripts were analyzed using the qualitative content analysis approach described by Mayring.

**Results:**

We interviewed 15 patient-GP dyads (i.e., 30 interviews). The interviews yielded three general aspects of living with and caring for somatic symptom disorders from the complementing perspectives of patients and their treating GPs: (1) The (undisclosed) patient side (partly invisible to the GP), (2) the shared part of the consultation in general practice, and (3) GPs’ management and care. Patients and GPs alike talked about consultations focusing mainly on communicational and relational aspects. Patients painted a picture of living with somatic symptom disorders, giving details about the relevance of personal situation, relationships, and their work situation for their symptoms and illness management. GPs’ focus lay on coordinating functions, and treatment and management strategies for patients with somatic symptom disorders.

**Conclusion:**

To establish a trusting relationship between patient and general practitioner, removal of time constraints for consultations and preoccupations for symptom management should allow for better insights of the general practitioners in patients’ lives. If we allow for time to let the patient-doctor relationship grow, mutual trust may ensue.

**German clinical trial register:**

ID: DRKS00011380, registration date: 1st December 2016.

**Supplementary Information:**

The online version contains supplementary material available at https://doi.org/10.1186/s12888-026-08750-2.

## Background

Regardless of their cause, persistent somatic symptoms (PSS) are distressing somatic complaints that last for several months or more [1]. In general practice, PSS are often the reason for consultations [2]. In many cases, PSS occur in the context of a somatic symptom disorder (SSD [1]). According to DSM-5-TR, SSD is defined by persistent physical symptoms that are associated with excessive attention, worry, anxiety or activity and lead to significant impairment in the person affected [3].

German general practitioners (GPs) estimate 7.7% of their patients to qualify for a diagnosis of a SSD [4]. Clinical consultations in primary care for patients with PSS strain GPs and patients equally [5, 6]. The strain originates from barriers in the diagnosis and treatment of the affected patients [7, 8]. Although we know that these barriers relate to patients, to GPs, and to their interaction alike [7], studies usually employ only either the GP perspective or, less frequently, the patient perspective.

The GP perspective yields the pertinent issue of the difficult patient [9, 10]. It sometimes includes cynical and stigmatizing undertones and GPs expressing their reservations and negative emotions towards patients with PSS [11]. GPs admit their own lack of knowledge about diagnostic [4, 12, 13] and treatment guidelines and their concern to miss serious somatic diseases [8]. Simultaneously they perceive patient pressure to provide symptom relief [8]. Patients with PSS face negative attitudes, poor communication and stigma when navigating the diagnostic trajectory and experience an ongoing uncertainty while transitioning from searching for a cure to learning to cope [14, 15, 15, 7]. Some patients are not able to accept the link between emotions and symptoms when introduced by GPs [16]. In view of this, it is not surprising that GPs assess aspects of possible mental illness not readily accepted by patients as part of the explanation of the clinical picture [7, 17]. In contrast, the patients assess that psychosocial burden is not taken seriously by the GPs [6]. It is an open question whether GPs and patients (interviewed separately) would refer to different consultation priorities focusing on what is most difficult for themselves. GPs might mostly remember ‘difficult patients’ as those unable to accept mental illness [6]. Meanwhile patients might mostly remember their disclosure of psychosocial burdens as unheard or unrecognized [18].

Presently, the two perspectives of primary care physicians and patients are still unconnected. While many studies exist [7] that look at the problem from a one-sided perspective, only asking GPs or patients, there seems to be a lack of studies investigating the phenomena from a dyadic perspective.

Extended knowledge about the dyadic processes of living with and caring for somatic symptom disorders could help to create support for both parties, the patients and the GPs. Earlier detection of patients with somatic symptom disorders by GPs leads to more adequate treatment. Patients’ earlier acceptance of the (preliminary) diagnosis and potential treatment approaches leads to less suffering and a heightened chance of living a good life even with a somatic symptom disorder [7].

Therefore, the aim of our study is to examine patients’ and GPs’ perspectives on patient histories to identify barriers to and potentially helpful aspects of communication, detection, care for and live with somatic symptom disorders. The research objective was to determine the impact of different psychosocial factors on diagnosing, accepting and treating somatic symptom disorders at the GP’s office from patients’ and GPs’ view.

## Methods

This article presents results from the qualitative content analysis of dyadic qualitative interviews (with patients and their GPs respectively) conducted during the mixed-methods project “Identification of barriers and difficulties involved in the process of diagnosing somatic symptom disorders in primary care” [19].

### Recruitment

We aimed at recruiting 16 patient-GP-dyads following a criteria-based purposive sampling approach [20]. Age and gender of the patients as well as symptoms either fulfilling ICD-10- or ICD-11-criteria are important variables concerning experiences of living with and caring for somatic symptom disorders [21]. Based on these criteria we intended to recruit a mixture of older (> 50 years) and younger (≤ 50 years) patients of both, male and female sex, fulfilling either ICD 10-criteria of somatoform disorders respectively DSM 5-criteria of somatic symptom disorder in order to maximize the variation in the accounts of our participants. German GPs use the ICD-10 [22] diagnoses from chapter F45 to classify patients with somatoform disorders, but revisions of this diagnoses in the ICD-11 (following the DSM 5 [23, 24]) were foreseeable. The assessment of the patients’ diagnoses by the GPs was relied upon for recruitment. Following the qualitative interview, the patients interviewed underwent a diagnostic interview to verify that they belonged to the intended patient group. However, the results of the diagnostic interviews did not lead to the patients being subsequently excluded, even though the diagnostic criteria were not always fully met.

German GPs have usually completed further training in basic psychosomatic care. The curriculum includes basic knowledge on psychosomatic conditions, diagnostics, verbal intervention techniques and therapy. All doctors providing GP care under the statutory health insurance scheme (“Vertragsarzt/-ärztin”) were eligible.

To include patient-GP-dyads we started recruitment by inviting GPs to be interviewed and recruit a patient following designated inclusion criteria.

In Hamburg andsurrounding areas 1150 GPs received an invitation (written information, reply sheet) via mail (09–11/2016). GPs who consented to take part in the study received patient inclusion criteria, an invitation for the patient including study information and a contact sheet (informed consent to be contacted by our study team). Patients interested in taking part in the study received a written confirmation of the scheduled interview date after a phone conversation discussing questions. Patients were interviewed after giving their written informed consent to be interviewed and for their GP to be interviewed. Additionally, GPs obtained the patients’ relief from their obligation of confidentiality before being interviewed. Recruitment proceeded more slowly than planned, so that, due to time constraints, recruitment had to be halted once 15 dyads had been enrolled in order not to delay the rest of the project.

### Data collection

Throughout data collection and analysis we followed a realist approach [25]. We conducted semi-structured qualitative interviews [26] using two different tailored but corresponding interview guides (see suppl. Material 1 and 2). Interview guides were developed based on previous research [7, 12, 27, 28] and the research questions. Interviews were conducted by NP, LR and KW. LR and KW received interviewer training. The first interview conducted by each interviewer was discussed within the interdisciplinary team and feedback was provided. Patients were interviewed in the interviewers’ office; GPs were visited in their practices. All interviewers introduced themselves to the patients (name, profession and affiliation) before starting the interview. Interviews were digitally recorded.

As discussing patient-GP-relationship and patient histories may bring up sensitive topics or topics that members of the dyad do not wish to share with each other [29], we chose to interview each dyad member separately and did not adapt interview guides with information given by the other [30]. This allowed interviewees to express their perspective, to discuss sensitive topics and voice criticism openly [31, 32]. Besides, dyadic interviewing can be considered as a method of triangulation enhancing the reliability of the data [31, 32].

### Transcription

All digital recordings were transcribed verbatim by a student research assistant following the semantic-content-based transcription rules established by Dresing and Pehl [33]. To protect the interviewees’ identity, all names were replaced by numbers and all potentially identifying details were changed. Transcripts were checked for accuracy by the respective interviewer.

### Data analysis

Interview data was analyzed by KK, ML, and NJP using the method of qualitative content analysis [34–36]. Qualitative content analysis aims [34] to describe a social phenomenon by breaking down the material into small units of analysis thereby reducing the amount of data while preserving the essential content of the data [35, 36]. We followed the approach of structuring qualitative content analysis by Mayring [35] deriving deductive categories from previous research [7] and adding inductive categories if needed. The scope of coded segments could range from part of a sentence to one or more paragraphs depending on the amount of data needed to understand the content and context of the aspect of interest. Categories and codes were described extensively in code-memos which were supported by concise quotes. All transcripts were coded by one of the analyzers and codings were controlled by a second analyzer regarding the classification of text passages into the categories of the category system. Discrepancies in coding were resolved by team discussion. Inductive thematic saturation of the material was assessed throughout the analysis and was deemed to have been reached towards the end of the analysis, as no new inductive categories emerged [37].

We chose to do ‘dyadic analysis’ (p. 1642, [31]) to identify overlap and contrast in patients’ and GPs’ data. Dyadic analysis includes a constant comparison between the findings in one group and the other and relating findings to each other. After analyzing the interviews from both groups separately we merged and contrasted accounts of patients and GPs to show similarities and discrepancy and create a dyadic version of the phenomenon “which is more than the sum of two individual versions” (p. 1642 [31]).

Preliminary results were presented in our interdisciplinary study group, at a conference and in an interdisciplinary seminar on qualitative methods led by NJP to check for intersubjective comprehensibility and to inform further analysis. Data was managed using MAXQDA 12 (Verbi GmbH).

### Researcher characteristics

ML is a male trained psychologist and post doctorate senior researcher. NJP is a female trained psychologist and post doctorate senior researcher, with comprehensive experience in conducting (dyadic) interviews as well as qualitative data analysis. KW is a female trained psychologist. LR is a female medical doctor.

## Results

We interviewed 15 patient-GP-dyads. Patients (female = 10, male = 5) were between 19 and 77 years old. Of these patients, eight (female = 5, male = 3) met the diagnostic criteria according to ICD-10, whilst two (female = 1, male = 1) met the diagnostic criteria according to ICD-11. The remaining five patients (4 female, 1 male) did not meet these diagnostic criteria, but all exhibited numerous persistent physical symptoms without any underlying conditions that could adequately explain them.

The GPs interviewed were either specialists in internal medicine (*n* = 4), specialists in general practice (*n* = 9) or “praktischer Arzt/Ärztin” (general practitioner, *n* = 2), aged between 35 and 66 and had between 1 and 26 years of professional experience at the time of the interview. Basic psychosomatic care was offered by thirteen physicians.

The interviews yielded three general aspects of living with and caring for somatic symptom disorders from the complementing perspectives of patients and their GPs. First, the (undisclosed) patient side which is partly invisible to the GP: living, working, and coping with somatic symptom disorders. Second, the shared consultation consisting of communication about symptoms and disease and being coined by communication style and the patient-physician-relationship. Lastly, management and care: GP’s treatment and management strategy, cooperation, and understanding of the somatic symptoms.

**Fig. 1:**
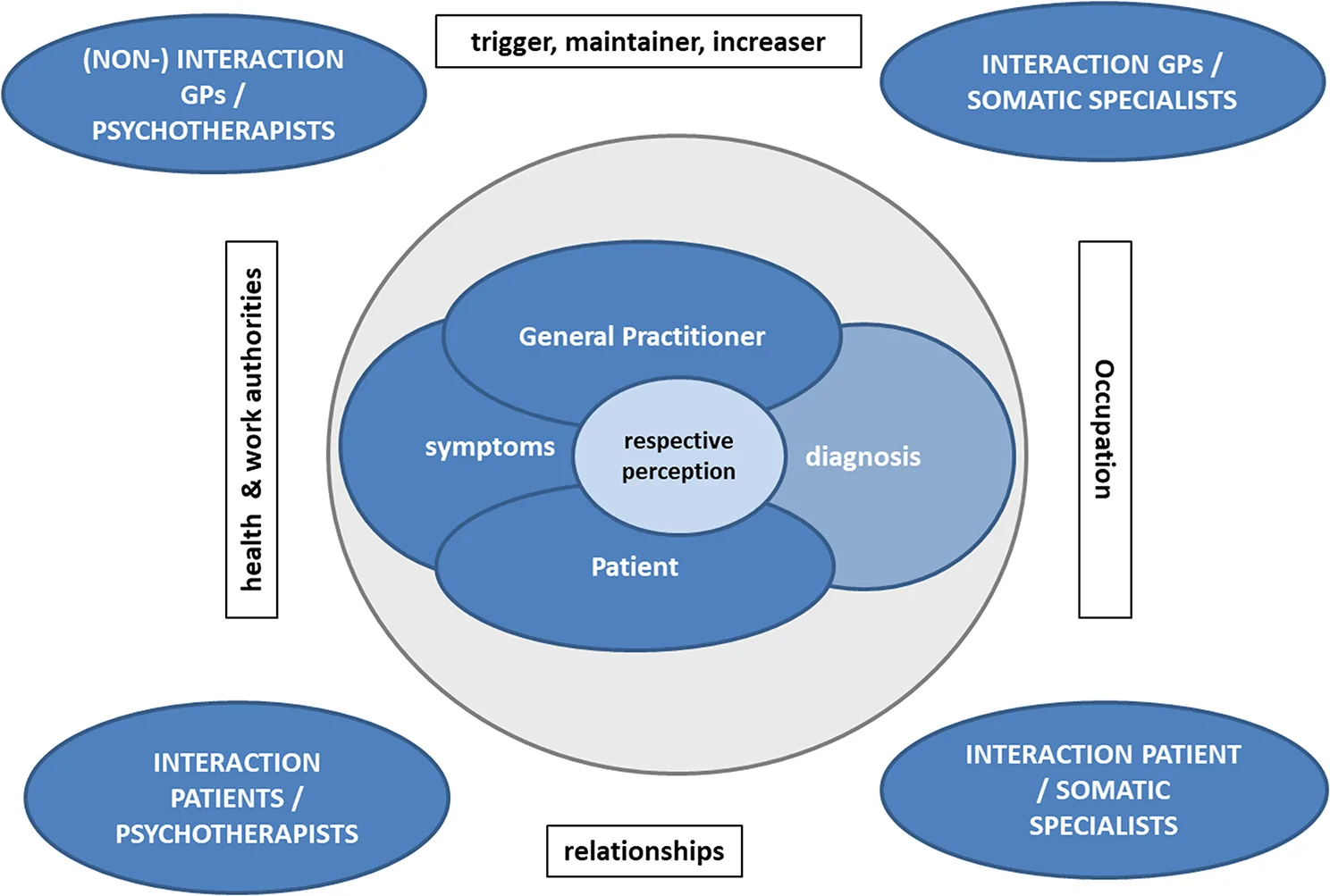
Context and influence factors of living with and caring for somatic symptom disorders

Interviewees described their experiences of living with and caring for somatic symptom disorders as influenced by several context factors. Figure 1 shows our model of context and influence factors.

The following sections reproduce the statements made by the people we interviewed regarding their symptoms, behavior and outlook on the world.

### Living with somatic symptom disorders

#### Patients’ situation and personal relationships

Interviewed patients often reported feeling alone with their problems. On one hand, patients, for example, felt alone with coping with trauma (e.g., loss of a close person), which often marks the beginning of the somatic complaints. On the other hand, a trigger for the start of complaints is not identifiable (neither for the GP nor for the patients). Perpetuating factors for somatic complaints could be manifold. For example, the patients’ idea of having to meet (high) external standards, causing them a high amount of stress. Conflicts with close ones, cold and distanced personal relationships or the burden of being surrounded by other chronically ill people needing support were mentioned. Talking about distressing situations is often impossible for patients. These strains entail two tendencies: the idea of having to get by alone and without help, and the realization that things cannot go on like this.

I was alone with my pain except for my [female] friend and the therapist, because my ex-husband didn’t want to hear about it. […] And in May [year X] I told my ex-husband, ‘If we don’t change anything in our lives, then in six months there’ll be a cut’. (female patient #5, par. 80)

I don’t know how to express it. Where the pain was so bad and I still wanted to be the functioning one. (female patient #9, par. 225).

[…] and the fact that my husband has to go to City E alone and I’m sitting there in the clinic [with my mother-in-law] triggered a rift, where I then also realized that even if I were at home now, I’m at the end of my tether psychologically. (female patient #8, par. 170).

Interviewed GPs reported to have only fragmentary insights into triggering and perpetuating factors of somatic complaints of their patients. GPs acknowledge that they would perceive patients’ psychological strain and the role of distressing social relationships. They often have their own explanation for patients’ complaints and express ideas for possible interventions. GPs see patients’ benefits from massive somatic symptoms, allowing them to withdraw when missing social acceptance. This may develop and increase over time. They spoke about comorbidities and symptom overlap with depression, anxiety, and fatigue. The interviewed GPs hypothesize that these comorbidities might have a reciprocal influence.

[…] the problem was, in her life she was the way she was/was wrong. And the way society wanted her to be, she could no longer be. […] And that had now been taken away from her and […] she developed enormous symptomatology. (GP of female patient #9, par. 42).

And those are the two things that are bothering him. Work and his father/ he hasn’t contacted his father in a year, right? He’s kind of avoided that now, so. Those are the two/the two things. (GP of male patient #3, par. 102).

#### Patient’s occupation

Patients and GPs alike highlighted continuation of work and medical certificates as central themes. Patients, furthermore, felt ambivalent regarding continuation of work or changing their workplace or profession. Patients talked about surrounding conditions, contacts with the employment agency, and potential vocational retraining. For example, the employment agency, being an external authority having expectations and making claims against patients, seemed to affect the doctor-patient-relationship. GPs reflected mutual influences of work-related factors and patients’ complaints and to what extent the complaints have consequences for the patients’ (ability to) work. They based their decision to issue sick leave certificates also on these reflections. However, patients and GPs draw an almost cynical picture talking about routinely issued sick leave certificates for satisfying the employment agency without having (agreed upon) a (psychotherapeutic) treatment regime for symptoms and impairments. GPs imparted that they were aware of their role as an assistant in this scheme but also reflected about their patients’ job re-entry. In fact, they felt that many patients wanted to work and to avoid sick leave.

I do it because that’s what he [the patient] wants. […] We haven’t really changed anything from the basis, from the root causes. Because he has never really undergone psychosocial, depth psychological treatment. (GP of male patient #1, par. 84).

[…] at the moment when the physical complaints are so much in the focus and in the foreground, that also entails a lot, that is, the ability to work was no longer there. (GP of female patient #8, par. 44).

Correspondingly, patients were ambivalent whether to continue or end their current employment or to withdraw permanently from work. It sometimes appeared as if they would maintain a malfunctioning system by suspending the decision for or against withdrawal. They feared unemployment because of somatic complaints and sick leave. Furthermore, they expressed self-chosen occupational strain and ideas of obligation to function on the job. GPs confirmed these ideas.

Of course I want to do it, but on the other hand, if I do it, […] how long will I do it? How long? Until the next hypertensive crisis? (…) […] Or until I fall off the chair at some point? (female patient #7, par. 298).

Doing a lot, a lot, a lot for others and always forgetting herself. […] I think she must not have been ill for ages before and goes to work with every flu-like infection. […] (GP of female patient #10, par. 24).

### Consultation in general practice

#### In-depth conversation, symptom management, and feedback on diagnostic tests

Both parties see in-depth conversations as helpful to build a relationship and for treatment. GPs reported lack of time and constant need to handle actual and shifting symptoms to prevent such conversations, holistic approaches and extensive anamneses. Patients, on the other hand, report past consultation reasons not being revived, unsuspicious test results not being communicated and consultations being reduced to handling actual symptoms.

No, I don’t actually go that far with her, because symptoms are always pushed to the fore again, so to speak, or come to the fore again. […] when she sits with it/ with a stridor in her breathing or with twitching, of course you must act first or at least rule out that it’s not really something dangerous, like that. […] (GP of female patient #1, par. 40).

Patients’ moaning about lacking feedback on test results was somewhat surprising as GPs report to use neutral test results as a starting point for conversations on psychosomatic roots of symptoms and to work with regular appointments with these patients.

[…] For example, when you want blood results, I don’t like to be told ‘If you don’t hear anything, then everything is fine’. I would like to get a call saying, ‘Everything is fine’, because that way I […] also have the feeling that maybe no one has looked at it. […] (female patient #3, par. 8).

#### Demands, challenges and stigma mark the consultation

While GPs described themselves as trying to avert stigmatizing patients, patients often reported experiences of stigma.

[…] ‘Find a pain therapist. It’s in your head.‘ (female patient #3, par. 193).

When you talk about it […] you have to be careful in your choice of words. So, the word psychiatry, psychiatrist is something you better avoid. (GP of female patient #2, par. 242).

GPs and patients reported two contrasting aspects of patients’ communication behavior. Both parties spoke of somewhat reserved and secretive patient behavior concerning impartation of information. GPs reported this behavior to affect information about the previous course of treatment and consultations with other physicians. Either way, both, patients and GPs reported mental health, psycho-social, and other information, for example, details about private and work life to be potentially relevant for diagnosis and treatment.

No, so from my past/, I mean what/ what should I tell her there/ […] no, I actually go there because of my complaints. (female patient #4, par. 124).

The interviewed GPs described patients as partly challenging, very vehement or demanding. Correspondingly, some patients explicitly confirmed this behavior.

The vehemence. The vehemence with which she brought up her respective things, yes? So, if/ it wasn’t somehow like it usually is. […] Pressure, everything quite extreme, yes? […] (GP of female patient #7, par. 72).

[…] I asserted myself in such a way that she gave me the appointment. […] Afterwards she also said, ‘But you’ve run me over here’. (female patient #7, par. 146–148).

Patients described themselves as demanding diagnostic tests, independently searching for information and handling medications, and initiating specialists’ (diagnostic) clarification and alternative treatment methods. They viewed their behavior as justified by their subjective illness. GPs described patients (often due to this exact behavior) as emotionally strenuous and needing much consultation time.

It was chaos. Mrs. [X] never came on her due date, always without an appointment with a high time requirement […] The assistants were already annoyed, and I always tried to keep things in order, and it was extremely tedious. […] (GP of female patient #7, par. 36).

While GPs confirmed patients’ adherence or compliance problems, they also described many of their patients as treatable and cooperative. With increasing extent and long-term nature of the GP-patient-contacts treatment relationships grow stronger and patients become easier to treat.

[…] but the treatment is going better now. When I tell her, “That’s too much thyroid hormone”, I see that next time she also picks up the right prescription dose and doesn’t say again ‘But I need 125 mg, otherwise I’ll feel bad’. (GP of female patient #7, par. 76).

#### Trusting relationships between patients and physicians

Both GPs and patients talked about difficulties establishing a trusting (treatment-) relationship. From the patients‘ point of view, sometimes several attempts are needed to find a GP who allows them to build mutual trust and a working treatment relationship. GPs confirm relationship-building to be more difficult and time-consuming and to create different challenges than with other patients. GPs see themselves often as important attachment figures for these patients.

It took me almost two years, after a long odyssey, to find the right doctor. (male patient #5, par. 24).

I also had the feeling that she (…) yes, [feels] in good hands here […]. But that she dares to come back here with the same old cheese [complaints] (laughs) without feeling unappreciated […]. (GP of female patient #2, par. 252)

With increasing trust, resulting from a long treatment relationship, it gets easier for patients to open themselves and for GPs to acknowledge this. Disruptions of trust result on patients’ side from the perception of not being taken seriously, not being seen in a holistic way and of the GP as not fulfilling all demands.

And then it got a bit down to the nitty-gritty, where she shed a few tears. She told us about her past, about her problems. That has only come about in the last few months. […] (GP of female patient #5, par. 20–28).

Patient-reported GP bafflement is often reflected in GPs subjective feelings of help- and powerlessness in relation to these patients.

Alarm and powerlessness at the same time, […] because she attacked me with her things, yes? And the order, the common thread, we couldn’t grasp it for a long time […]. (GP of female patient #7, par. 74–76).

If the GPs perceive that the patients focus exclusively on possible somatic causes of the symptoms, they sometimes opt to follow a somatic approach to treatment.

Yes, of course, if this is met with resistance and rejection again and again, it is a difficult situation. Then it’s not easy and at some point the repression mechanism might set in. That I focus on physical complaints. […] (GP of female patient #8, par. 50).

### Caring for somatic symptom disorders

#### GP’s management strategies – coordination and cooperation

Patients‘ and GPs‘ descriptions of patient histories mostly corresponded to each other. Both assigned at least some of the symptoms to underlying somatic disorders. Patients’ accounts seldom reveal whether these explanations rely on subjective lay theories or on medically established evidence.

Because she (…) actually described a lot of different symptoms that also kept her very busy. She was also very worried about them. But there are no really objective findings. (GP of female patient #10, par. 18).

And then he also said ‘OK’ and took blood and I had an elevated ANA [antinuclear antibodies]-titre. And then he said ‘Okay, many of your complaints could really also come from a rheumatic disease’. (female patient #6, par. 31).

While patients reported less on their own consultation behavior or their strategies to navigate in the health system, the GPs’ account focused on these themes and patients’ handling of their symptoms and disorders.

[…] She was running from/to somebody, somehow disorderly, bringing some kind of findings, wanting something, which was not comprehensible to me at all, because she was more or less out of control, also in terms of consulting specialists. […] (GP of female patient #7, par. 38).

Patients and GPs, discussed the connection between patients’ symptoms and complaints, and mental (co-)morbidities. Patients described avoidance behaviors, and other self-regulation strategies to ensure that symptoms are kept within limits or do not worsen.

No, at the moment I think the problem is that she is trying to process too many things in the context of this trauma therapy, and I think that at the moment this rather activates symptoms and intensifies the symptoms. (GP of female patient #5, par. 146).

No, the only thing that really helps is to stay at home and cancel the plans, so to speak, and (…) then calm returns immediately. Because the thing that made me nervous in the first place is no longer there. […] (male patient #2, par. 189).

GPs rather focused on patients’ excessive preoccupation with their symptoms and dramatizing presentation in consultation. This sometimes leads GPs to carry out diagnostic and treatment measures they deem unnecessary. Not all patients are doctor shoppers from the GPs’ point of view, whereas they admit that they can never tell.

From the scene she delivers to me here in the consulting room, scenically. […] Highly under pressure, highly fixated on symptoms and a discrepancy between […] findings and condition, […] (GP of female patient #7, par. 56).

Yes, they are also not very aware […] that their somatic disorders are not of somatic origin, and they just don’t see that, but then they often don’t come back, do they? So, you can try without end, they then make a round trip. (GP of male patient #5, par. 96).

Patients and GPs talked positively about the coordinating function of the GPs, which patients circumvented sometimes. Especially to be able to make use of existing cooperations between GPs and other specialists and psychotherapists, sometimes even in the GPs’ practice, is positively viewed by patients. GPs lamented on insufficient cooperation with psychotherapists, especially regarding the flow of information from the psychotherapists back to the GP. The possibility of regular exchange was desired and seen as potentially relieving.

#### Treatment of somatic symptom disorders

The patients described strategies for medical treatment and alternative approaches to therapy. In contrast, the GPs rarely mentioned medical treatment and complementary medicine. Patients listed an arsenal of medicine used to treat their symptoms. Although expecting positive effects of medication, the patients also describe their ambivalence regarding possible side effects and addiction.

Self-medication, okay, I’ll just take ibu[profen]. And at first, it helped, but now it doesn’t work at all. Now we are at Novalgin [Metamizol] drops and Berlosin [Metamizol] tablets. Berlosin, yes, okay, but only to a certain extent, depending on the pain at the time. And Novalgin, yes, I run around like I’m drunk. I can’t always do that either, eh? (male patient #5, par. 56).

In patients’ words, the demand for medication almost appears like a negotiation with the GP, in which also acute needs of medicine “when one feels sore” are possible. Admittedly, GPs reluctantly prescribe medication if no physical disease is present.

When I was given tablets, I first checked the side effects and then stopped taking them. […] and when I noticed that I was getting a bit restless again and that dizziness or something else was occurring again, I went and said, ‘I need another one.’ (female patient #2, par. 242).

Furthermore, patients mention alternative therapies including relaxation techniques, acupuncture, yoga, or mindfulness exercises. It seems that these procedures could also lead patients to ignore the psychological part of their complaints – a practicable way to avoid the confrontation with a possible psycho-social burden.

No, I had already been to a non-medical practitioner before […] But she also mainly tried to bring the intestines back into balance with small homeopathic medicines and (.) didn’t really deal with the psyche. (male patient #2, par. 105).

The GPs also reported about patient demands when something is pending or after relapses and their strategies of watchful waiting and ruling out serious physical conditions. With persistent somatic symptoms, however, these demands sometimes get out of control, leading the GPs to speak of emergency consultations.

It is, but it/ is but with patients who are very anxious or very agitated, you can forget your control mechanisms […], so you can forget them. (GP of female patient #2, par. 25).

Despite their frustration about the lack of improvement with these patients, the GPs, nevertheless, quasi-contracted the acceptance of sickness with the patients. GPs traced some treatment successes back to their strategy of reassurance and availability as contact persons. Furthermore, they also encourage patients to reflect on the idea of a psychosomatic cause of somatic complaints. Thereby, the outworn reassurance that there is no somatic cause for the symptoms is replaced by more tangible explanations.

[…] to open up the inner understanding that there are other things going on with her than just her derailed blood pressure or her headaches or any intolerances, yes? […] I have known her for a long time, there have been many different episodes. […] But at those times she was not at all open to any intervention that would have touched in any way on anything other than ‘It’s the hormones now’ or ‘It’s the surgery’ or ‘It’s something physical’ that she was latching onto […]. (GP of female patient #7, par. 22).

The impetus for psychotherapy emanates from the GP and the effort to start it from the patient. For example, the patient is expected to directly contact therapists or to work through a list of help service telephone numbers. Hereby, the frustration of a long wait arises until therapy begins. This was noticed likewise by patients and GPs. Accepting a particular therapy offer is sometimes complicated, whereby the patient’s friends and acquaintances as well as the GP’s personal contacts play a pivotal role. Although the GPs would like to monitor the progress of therapy, they reported not being involved enough.

[…] I was very lucky. She wasn’t in the telephone book or in the newspaper but only got her patients by word of mouth. And so, I waited three weeks and then I already had an appointment with her. (female patient #2, par. 155).

GPs described the transition from persistent physical symptoms to a diagnosis of a mental disorder as problematic. Particularly, the label mental disorder would bear problems. The patients reluctantly accept the association between somatic complaints and psychological factors. GPs note that an unhelpful dualistic view of body and mind is the core problem. Therefore, GPs appraise early contact with psychotherapists.

And so, we have to have the time to recognize that [unclear physical complaints]. We must have the time to discuss it and then it should go to a psychotherapist fairly quickly. […] (GP of male patient #3, par. 272).

[…] it [psychotherapy] has to be done in a much more […] in a long-term way. […] So, that you lay the foundation for it or really have a long conversation, […] but not the therapy of a psychotherapist or psychosomatic [physician] or psychologist here in the family doctor’s practice, which I can’t render, no. (GP of female patient #1, par. 118).

However, even if this succeeds, according to the GPs, the lack of therapy options remains and patients may not personally match with the therapist.

[…] the hurdle of looking for someone to open up to is of course huge and the hurdle is much bigger when you are handed a list ‘You can call here everywhere’, no? And then not even knowing/ well, not even having the feeling ‘I can choose who suits me. I just must take what comes along’. And I think that is often the biggest hurdle. (female patient #1, par. 136).

## Discussion

### Summary

In our study patients and GPs described living with and caring for somatic symptom disorders from complementary perspectives. The two perspectives merge into a less idiosyncratic view of the situation in general practice. Patients and GPs alike talked about the consultations focusing mainly on communicational and relational aspects. Patients painted a picture of living with somatic symptom disorders, giving details about the relevance of personal situation, relationships and their work situation for their symptoms and illness management. GPs focus lay on their coordinating functions and their treatment and management strategies for patients with somatic symptom disorders.

We conclude that the working alliance between patients and GPs requires mutual trust and acceptance of patients’s symptoms and living situation. Mutual trust is established when known diagnostic and treatment barriers are overcome and patients express not only their physical symptoms but also their psychosocial distress. GPs in our study seemed to be well prepared and open to engaging with this information. Acceptance is required on both sides; that is, patients accepting that psychosocial distress may play a role in the emergence and maintenance of physical symptoms and GPs accepting that it is the whole patient living with somatic symptom disorder who needs care in all bio-psycho-social respects. Not surprisingly, offering adequate care requires longer consultation time and continuity over longer time periods, which were repeatedly expressed by GPs as two main desiderata.

### Implications of findings in context of existing research

The importance of patient-physician connectedness and personal continuity of care for (positive) primary care (outcomes) is well-known, e.g. [38–40]. Even though interviewed patients and general practitioners alike expressed wishes for a trusting relationship as a foundation of care patients did not regard their GPs as the right addressee for disclosing information about their emotional or mental wellbeing. Other studies also showed that about one third of patients in primary care do not want to disclose psychological problems to their GPs and believe that primary care consultation is the wrong place for this topic [41]. Treufeldt & Burton [15] showed the need for effective stigma reduction in consultations on persistent physical symptoms and functional disorders. Also, destigmatizing mental illnesses and educating patients about the appropriateness to discuss mental health issues in general practice could improve primary mental health care [41]. GP training could improve patient satisfaction [42] and outcome [43].

Interviewed patients disclosed ambivalent feelings towards (quitting) their work and potential unemployment. They sometimes view their work as a perpetuating factor, a view they often share with their GPs. Having “invisible” symptoms can be an additional burden to the sickness leave - and its possible consequences [44]. GPs reflected on their role as providers of sick leave certificates which they issue sometimes with reluctance and sometimes generously. Aamland et al. [45] showed that the probability of a doctor issuing a sick leave certificate increased with doctors knowledge of the patient, sympathy and trust. This and our study results reflect the challenges GPs face when dealing with the issue of sick leaves for patients without a defined diagnosis, for example, difficult conversations about (dis-)advantages of sick leaves and the potential risk of losing the patient to another GP more willing to issue sick leave certificates [46]. Related studies showed that interventions directed at doctors do not tend to lower sick leave [45].

Our interviewees, patients and GPs alike, underscored the importance of longer consultations with adequate time to discuss symptoms and potential diagnostics and the meaning of its results. Patients with PSS repeatedly emphasized the significance of a good physician-patient-relationship and feeling to be taken seriously (e.g [47, 48]). Either way, our interviewees (patients) described themselves sometimes as reluctant to disclose information or very demanding and challenging, which is supported by GPs accounts in the interviews, in focus groups conducted in this project [not described here, see [12] ] and other studies [7]. The desire to be heard is a recurring theme in research on somatic symptom disorders [2, 9]. GPs’ assessment of patients’ reasons for frequent attendance can reduce consultation frequency [49], but takes time. In the consultations, however, the general practitioners get only fragmentary insights in the patients’ lives and envision their own explanations about how psychosocial burden and somatic symptoms relate. Patients with somatoform type symptoms interviewed by Houwen et al. [47] underline the importance of continuity of care and the GP knowing their personal background as well as the proper management of their somatic symptoms. The authors emphasize the importance of the therapeutic relationship and conclude that GPs should invest time and energy to explore individual patients’ backgrounds and contexts, communicate at eye-level and spend more time on the exploration and management of somatic symptoms. Our interviewees also supported the notion of the GP as coordinator of care, but this is complicated by theories of illness differing between GPs and patients and sometimes rather uncoordinated service utilization behavior and doctor-shopping. This is not new and supported by Murray et al.’s review [7]. Norton et al. [50] found that patients with mental illnesses saw different doctors more often when they were dissatisfied with their previous GP.

Over time, we observe a promising development, nevertheless. Accounts from the 1990s express stigma with labels such as ‘difficult patient’ or the tenet that patients would maintain power over their GPs [51–53]. This view shifted in the 2000s towards understanding patients‘ voices - how they express symptom burden - and towards empathy with the patients‘ situation [6, 54]. The introduction of SSD in comparison to the former somatoform disorder embodies the idea of moving psychological burden into the focus of diagnostic attention. Talking with patients could now be grounded in rational reasoning using ideas of symptomatic facts, hypothesized causes and mechanisms of how symptom burden emerges [55–57]. Contrasting this optimistic stance with our results, we rarely observed rational reasoning between patient and GP, but observed pragmatic case management as the main strategy. Patients and GPs build their working alliance on trust and the acceptance of the patients’ private situation [58–60].

### Strengths and limitations

To our knowledge, this is the first study to incorporate patient-GP-dyads to include and compare the two sides of the medal in this handling and diagnosing process. This triangulating approach [61] creates the advantage of inter-related data giving a deep holistic picture of the process.

Not all patients invited by GPs matched the proposed inclusion criteria described above regarding their diagnoses according to ICD-10 and ICD-11. This likely reflects the usual GP-patient-relationship in primary care being more focused on the whole person and ‘reasons for encounters’ and the challenges associated with diagnosing and managing these disorders [12, 62]. Findings might have been even more pronounced with exact matches. Either way, we think that our data draws a realistic picture of the situation of patients with somatic symptom disorders in primary care and can be cautiously extrapolated to relevant patient populations.

GPs might have chosen patients they were more attached to or just being more prominent in their minds. We also cannot rule out that patients with a more positive relationship with their GPs were more willing to take part in our study, distorting the picture. The criterion-based sampling process aiming for maximum variation in the interviewees account might have reduced these limitations and we consider thematic saturation [37] reached as no more inductive categories emerged from the data nearing the end of data analysis.

## Conclusions

The relevance of our findings lies in the possibility of viewing the same patient with a somatic symptom disorder from different, but related perspectives. Many studies focus either on the professional perspective of the GP (e.g, [9, 10, 15, 63]), or the perspective of the affected patient (e.g, [14, 15]). The use of dyadic separate interviews increased the depth of the findings on living with and caring for somatic symptom disorders. In a next step, observations of consultations not only with GPs, but also other specialists followed by joint dyadic debriefing sessions and case conferences would be even more interesting to deepen the understanding of these processes.

At the heart of successful management and treatment of somatic symptom disorders lies a trusting relationship between patient and general practitioner. This relationship needs time to develop [64], because barriers impede mutual trust. Patients and general practitioners alike want this trustful relationship, but are often bound in excessive occupation with symptoms or time constraints (see also [47, 65]). Consequently, in depth conversation as a vital factor to develop trust is neglected. Patients are reluctant to disclose psychosocial burden and feel stigmatized. GPs feel powerless when it comes to providing care and engaging with patients, as acute care is focused on treating symptoms – a situation that creates further obstacles. An improved reimbursement of extended consultations and talking medicine [66] and increasing the status of person-centered clinical work [67] would help to improve the status quo of living with and caring for somatic symptom disorders.

## Supplementary Information

Below is the link to the electronic supplementary material.

Supplementary Material 1

Supplementary Material 2

## Data Availability

The datasets generated and analyzed during the current study are not publicly available due to the assurance to participants that the full raw data would not be shared publicly, and that all attempts would be made to maintain confidentiality, but are available from the corresponding author upon reasonable request.

## Abbreviations

DSM-5: Diagnostic and statistical manual of mental disorders fifth edition
GP: General practitioner
ICD-10: International classification of diseases 10th Revision
PSS: Persistent somatic symptoms
SSD: Somatic symptom disorder
