# Supplementary material for: Living with and caring for somatic symptom disorders: a qualitative interview-study with patients and GPs using a deductive-inductive content analytic approach
Source: BMC Psychiatry. 2026 Oct 13;26:785. doi: 10.1186/s12888-026-08750-2 (PMC13660235; doi:10.1186/s12888-026-08750-2)
Supplement: Supplementary file 1 [file 12888_2026_8750_MOESM1_ESM.doc]

Introductory information on interviewer and study

Any questions left before we start?

**The patient**

First of all, please describe your relationship with Mr./Mrs. [name].

You have chosen Mr./Mrs. [name] for our study on handling of patients with unclear and wearing complaints. Why?

Please describe Mr./Mrs. [name]’s patient history regarding his/her unclear and wearing complaints and his/her related sorrows. From the first appearance of the symptoms until today.

Would you say that Mr./Mrs. [name] has a somatoform disorder? If so: How do you know?

If not: You have told me that Mr./Mrs. [name] shows excessive sorrow or engagement with his/her wearing complaints. How did you know that this behaviour might be clinically significant?

What helped you with diagnosing and caring for Mr./Mrs. [name]? Which resources, for example guidelines, did you consult?

Was there a point in time when you had to choose between different options?

To which extent where sick certificates of incapacity for work relevant?

Do you think that there are any sustentative factors in regard to Mr./Mrs. [name]’s illness?

Are there any social sustentative factors?

Do you think that Mr./Mrs. [name] profits somehow from his/her complaints?

Have you been under the impression that psychological factors play a role concerning these complaints? If so: How did you address this issue with Mr./Mrs. [name]? How did you try to communicate this to him/her?

In your opinion, how does Mr./Mrs. [name] explain his/her complaints and sorrows?

Do you have the feeling that you could help Mr./Mrs. [name]? How? Why not?

What would have helped you in caring for Mr./Mrs. [name]? Any wishes?

Is the financial compensation for basic psychosomatic care high enough to care adequately for patients like Mr./Mrs. [name] or is a different kind of compensation needed?

How could these patients‘ journeys through the health care system possibly be shortened?

**Conclusion**

Is there anything else concerning diagnosis and treatment of somatoform diseases you want to add?
