# Supplementary material for: Living with and caring for somatic symptom disorders: a qualitative interview-study with patients and GPs using a deductive-inductive content analytic approach
Source: BMC Psychiatry. 2026 Oct 13;26:785. doi: 10.1186/s12888-026-08750-2 (PMC13660235; doi:10.1186/s12888-026-08750-2)
Supplement: Supplementary file 2 [file 12888_2026_8750_MOESM2_ESM.doc]

Introductory information on interviewer and study

Any questions left?

First of all, please tell me something about yourself. How old are you and what do you do for living? Are you in a relationship at the moment? Do you have any children?

**Start**

How long are you Dr. [name]’s patient now?

What is your assessment of your relationship to Dr. [name]? How satisfied are you with Dr. [name]?

**Complaints**

Now I would like to ask some questions about your patient history in general. Please answer as detailed as possible.

You had impairing complaints for some time now. Please tell me about these complaints.

Which kind of fears or sorrows do you harbor concerning your complaints?

Do you talk with persons other than Dr. [name] and other physicians about your complaints and sorrows? What do these persons tell you concerning your complaints?

Is there anything concerning your complaints you don’t want to talk about with Dr. [name]?

Is there anything concerning your complaints that is easier to talk about with Dr. [name]?

Why is that so?

**Diagnosis and treatment**

You have told me about your symptoms and sorrows. How were your fears and sorrows handled? Please tell me the whole story from the onset of the symptoms till today.

How do your complaints and sorrows influence your daily live?

Were there times in the past when you weren’t able to work because of your complaints? If so, how often and how long?

What did the GP tell you about the origin of your complaints?

What did other physicians tell you about the origin of your complaints?

Did you agree with this explanation? What do you think is the origin of your complaints?

Please tell me what will happen from now on?

Is there something that you would have expected from Dr. [name] in the past?

**Conclusion**

Is there anything else you would like to add to your patient history and everything else we have discussed today?
